# Association analysis between *CD14* gene polymorphisms and peri‐implantitis susceptibility in a Chinese population

**DOI:** 10.1002/iid3.1230

**Published:** 2024-04-17

**Authors:** Jie Li, Xiao Qiao, Jin Shang

**Affiliations:** ^1^ Department of Stomatology Yangzhou Hospital of TCM Yangzhou China; ^2^ Department of Oral and Maxillofacial Surgery Taizhou Stomatological Hospital Taizhou China; ^3^ Department of Stomatology Affiliated Hospital of Yangzhou University Yangzhou China

**Keywords:** *CD14*, peri‐implantitis, polymorphism, susceptibility

## Abstract

**Objective:**

The goal of the study was to examine the genetic correlation of cluster of differentiation 14 (*CD14*) gene polymorphisms with peri‐implantitis (PI) predisposition in a Chinese Han population.

**Methods:**

In the case‐control study, blood samples were collected from PI patients and healthy individuals (*n* = 120/group), who were admitted to the Affiliated Hospital of Yangzhou University from 2021 to 2023. One‐way analysis of variance (ANOVA) was applied to compare differences of continuous variables among different groups. Genotype and allele distributions of *CD14* gene rs2569190 and rs2915863 polymorphisms were analyzed between groups via *χ* ^2^ test.

**Results:**

A high percentage of rs2569190 GG genotype or G allele carriers were identified in PI group compared with control group (*p* < .01). Rs2569190 GG genotype carriers had high risk to develop PI (odds ratio: 2.545, 95% confidence interval: 1.257–5.156, *p* = .009). The rs2569190 AA genotype carriers had the lowest values of gingival index, plaque index, calculus index, peri‐implant pocket depth, and clinical attachment level, which were the highest in cases with GG genotype.

**Conclusion:**

Rs2569190 polymorphism of *CD14* gene was significantly associated with PI predisposition in the Chinese Han population, and the GG genotype and G allele were risk factors for the development of PI.

## 1 INTRODUCTION

Peri‐implantitis (PI) is a chronic inflammatory disease, which initiates in the soft tissue and then progressing to the hard tissue surrounding dental implants. The incidence of PI is becoming more and more prevalent, leading to loss of osseous support and potential loss of the implant if not identified early in the process.^1^ Epidemiological studies have shown that approximately 30% of patients who receive dental implants would develop PI.^2^ But no effective therapeutic strategies have been explored against PI. PI is one of the most common complications of implant therpy, eventually leading to the loss of the functioning osseointegrated implants.^3^ Thus, PI has been considered to be the leading cause of late implant failure along with occlusal overload.^4^ As a multifactorial disease, it can be regulated by pathogenic microorganisms and host inflammatory immune response. Various complex interactions including host factors, genetic and environmental factors are involved in the pathogenesis of PI.^5^

Cluster of differentiation 14 (CD14) acts as a multifunctional high‐affinity receptor for the binding of endotoxins, lipopolysaccharide (LPS). It is involved in primary immune and inflammatory responses.^6^, ^7^ Additionally, LPS has been reported to be associated with the deterioration of gram‐positive bacteria induce.^8^ CD14 can proportionally regulate the subsequent production of pro‐inflammatory cytokines, eventually resulting in LPS‐stimulated bone resorption.^7^, ^9^ The CD14 expression has been determined to be involved in various pathological states, including periodontal diseases.^10^ Vijaya et al.^11^ have also reported a significant association between elevated levels of CD14 and human periodontitis. The human *CD14* gene is located on chromosome 5q31, and numerous single nucleotide polymorphisms (SNPs) have been detected in *CD14* gene, which can mediate the protein's function. Recently, the relationship between *CD14* gene polymorphisms and PI predisposition has been presented by Rakic et al.^12^ The ‐1619A/G (rs2915863) and ‐159C/T (rs2569190) are two common SNP in the promoter region of the *CD14* gene, and they are associated with altered levels of CD14.^13^ Previously, the two SNPs has been reported to be associated with the susceptibility of sepsis.^14^ Moreover, rs2569190 has been determined to be related to PI predisposition in Southeastern Europe Caucasians.^12^ However, their association with PI has not been evaluated in the Chinese Han population.

Therefore, the goal of this study was to examine the association of *CD14* gene rs2569190 and rs2915863 polymorphisms with genetic susceptibility to PI in a Chinese Han population, which will be beneficial for the exploration of the pathogenesis of PI.

## 2 MATERIALS AND METHODS

### 2.1 Subjects

A total of 240 individuals were enrolled in this case‐control study, including 120 patients with PI and 120 individuals with healthy implants. Cases in the PI group met the following inclusion criteria: (1) had at least one implant with the loading period longer than 12 months; (2) suppuration occurred on probing accompanied by bleeding and the probing depths greater than 5 mm; (3) one or more area presents crestal bone loss around the implant based on the Radiographic results, and at least two edges were exposed; (4) no occlusal trauma in the implant. The inclusion criteria of control group: (1) no history of periodontitis and clinical symptoms of periodontitis; (2) periodontal pocket depth is less than 3 mm; (3) no radiological findings of bone resorption. Exclusion criteria: (1) patients took immunological agents and antibiotics in the past 3 months; (2) patients undergoing orthodontic treatment; (3) patients with routine health problems such as hepatitis, diabetes and chemical therapy; (4) women during pregnancy and lactation. The patient's medical history was asked in detail, and their age, gender, body mass index, smoking history, drinking history, diet, and lifestyle habits and other basic information were recorded.

This study design was approved and consented by the Ethics Committee of Affiliated Hospital of Yangzhou University. Each participant signed and submitted a written informed consent. All individuals involved in this study were Chinese Han population and had no blood relationship with each other.

### 2.2 Observation index

Before treatment, Williams periodontal probe was used for the oral examination, and gingival index, plaque index, calculus index, peri‐implant pocket depth (PPD) and clinical attachment level (CAL) were recorded.

### 2.3 Genotype identification

Approximately 10 mL of venous blood was drawn from each subject into a vacuum tube containing anticoagulant EDTA. DNA extraction kit (Tiangen Biochemical Technology Co., Ltd.) was applied for extraction of genomic DNA. Then the concentration and purity of the DNA samples were detected, and stored at −80°C for testing.

The target fragments were amplified by polymerase chain reaction, and the total volume of the amplification reaction was 40 μL, including 20 μL premix, 10 μL DNA template, 1 μL upstream primer, 1 μL downstream primers, and 8 μL distilled water. The reaction conditions were denatured at 98°C for 10 s, annealed at 63°C for 5 s, and extended at 72°C for 10 s, a total of 40 cycles were accomplished. After the reaction, the purified products were collected and sequenced by ABI 3500DX sequencer (ABI Corporation).

### 2.4 Statistical analysis

SPSS 21.0 software was used for statistical analysis. For continuous variables, the data distributions were first evaluated for normality by the Kolmogorov–Smirnov test, and all variables did not conform to a normal distribution. So the data were expressed as median with interquartile range. The categorical variables were expressed as number and percent (%). The goodness‐fitting *χ* ^2^ test was used to analyze the Hardy–Weinberg equilibrium. One‐way analysis of variance (ANOVA) or *χ* ^2^ test was used to compare differences in general population data, periodontal clinical indicators and genotype distribution. Bilateral *p* < .05 was considered statistically significant.

## 3 RESULTS

### 3.1 General characteristics of the study population

This case‐control study was performed based on 240 patients receiving dental implants, among them 120 cases underwent PI while the remaining 120 individuals did not (Table 1). The control group consisted of 84 males (51.85%) and 78 females (48.15%) with the mean age and SD equal to 42.44 ± 5.92. Among the PI group, 89 (54.94%) cases were males and 73 (45.06%) individuals were females. The differences of both age and gender between the two groups were found nonsignificant (*p* > .05). The history of alcohol consumption, smoking and periodontitis also differed with no significant difference in the two groups (*p* > .05). The mouth washing habits, including frequencies of brushing, dental floss, and mouth washing had no prominent difference between the HC and PI groups (*p* > .05). Significant differences were found in respect to the periodontal status, it was shown that PI cases had high values of gingival index, plaque index, calculus index, PPD, and CAL (*p* < .05). But the platform type and position, per‐implant phenotype exhibited no highlighted discrepancy between the HC and PI groups (*p* > .05).

**Table 1:** General characteristics of the study population.

Indicators	HC (*n* = 120)	PI (*n* = 120)	*p* Value
Age (years)	44 (38, 49)	43 (39, 48)	.607
Gender (*n*/%)			.578
Male	84/51.85	89/54.94	
Female	78/48.15	73/45.06	
Alcohol consumption (*n*/%)			.910
Yes	65/40.12	66/40.74	
No	97/59.88	96/59.26	
Smoking (*n*/%)			.823
Yes	75/46.30	73/45.06	
No	87/53.70	89/54.94	
Periodontitis (*n*/%)			.059
Yes	73/45.06	90/55.56	
No	89/54.94	72/44.44	
Tooth loss reason (*n*/%)			.369
Periodontitis	71/43.83	83/51.23	
Deep caries	2/1.23	1/0.62	
Trauma	89/54.94	78/48.15	
Brushing daily (*n*/%)			.754
1–3 times	137/84.57	139/85.80	
More than 3 times	25/15.43	23/14.20	
Dental floss daily (*n*/%)			.231
Yes	63/38.89	67/41.36	
No	30/18.52	19/11.73	
Infrequent	69/42.59	76/46.91	
Mouth washing daily (*n*/%)			.363
Yes	48/29.63	60/37.04	
No	37/22.84	32/19.75	
Infrequent	77/47.53	70/43.21	
Periodontal status			
Gingival index	1 (1, 1)	2 (2, 3)	<.001***
Plaque index	1 (0, 1)	2 (1, 2)	<.001***
Calculus index	0 (0, 0)	0 (0, 1)	<.001***
PPD (mm)	2 (1, 2)	5 (5, 6)	<.001***
CAL (mm)	1 (1, 1)	5 (4, 5)	<.001***
Platform type (*n*/%)			.317
External hex	76/46.91	68/41.94	
Internal hex	24/14.81	37/22.84	
Morse cone	52/32.11	49/30.24	
Others	10/6.17	8/4.94	
Position (*n*/%)			.055
Anterior region	104/64.20	87/53.70	
Posterior region	58/35.80	75/46.30	
Per‐implant phenotype (*n*/%)			.149
Thin	89/54.94	76/46.91	
Thick	73/43.45	86/53.09	

Abbreviations: CAL, clinical attachment level; HC, healthy controls; PI, peri‐implantitis; PPD, peri‐implant pocket depth.

*** High statistical difference with the *p*‐value less than .001.

### 3.2 Frequency distribution of *CD14* gene rs2569190 and rs2915863 genotype and allele in HC and PI groups

According to the Hardy–Weinberg exact test results, the included two study groups were all in equilibrium, with a *p*‐value of more than .05 (Table 2). Based on the *χ* ^2^ test results, significant correlation between *CD14* gene rs2569190 polymorphism and PI susceptibility was found (*p* < .05), and there was a high risk of PI for GG genotype carriers (OR = 2.545, 95% confidence interval [CI] = 1.257–5.156). The correlation of rs2915863 genotypes with PI showed no statistical significance (*p* > .05). Moreover, the same statistical results were gained for rs2915863 allele distribution (*p* > .05). The results implied that *CD14* gene rs2915863 polymorphism was not related to susceptibility to PI.

**Table 2:** Frequency distribution comparision of *CD14* gene rs2569190 and rs2915863 genotype and allele in HC and PI groups based on *χ* ^2^ test.

Genotype/allele	HC (*n* = 120)	PI (*n* = 120)	*χ* ^2^	OR (95% CI)	*p* Value
rs2569190					
AA	48 (40.00)	33 (27.50)	‐	1 (Reference)	‐
AG	52 (43.33)	52 (43.33)	1.572	1.46 (0.81–2.62)	.210
GG	20 (16.67)	35 (29.17)	6.869	2.55 (1.26‐5.16)	.009**
A	148 (61.67)	118 (49.17)	‐	1 (Reference)	‐
G	92 (38.33)	122 (50.83)	7.589	1.66 (1.16–2.39)	.006**
*P* ^HWE^	.361	.145			
rs2915863					
TT	28 (23.33)	20 (16.67)	‐	1 (Reference)	‐
TC	50 (41.67)	49 (40.83)	0.795	1.37 (0.68–2.75)	.372
CC	42 (35.00)	51 (42.50)	2.197	1.70 (0.84–3.44)	.138
T	106 (44.17)	89 (37.08)	‐	1 (Reference)	‐
C	134 (55.83)	151 (62.92)	2.496	1.34 (0.93–1.93)	.114
*P* ^HWE^	.089	.171			

Abbreviations: AA, adenine–adenine; AG, adenine–guanine; CC, cytosine–cytosine; CI, confidence interval; GG, guanine–guanine; HC, healthy controls; HWE, Hardy–Weinberg equilibrium; OR, odds ratio; *p* ^HWE^ means the *p*‐value of HWE; PI, peri‐implantitis; TC, thymine–cytosine; TT, thymine–thymine.

** Significant difference with the *p*‐value less than .01.

### 3.3 Periodontal status in patients with *CD14* gene rs2569190 and rs2915863 polymorphisms

According to *CD14* gene rs2569190 genotype, all PI patients were divided into three group, namely, AA group, AG group, and GG group. Then the mean values of gingival index, plaque index, calculus index, PPD, and CAL were compared to assess the periodontal status of PI patients with different rs2569190 genotypes. As shown in Table 3, the AA genotype carriers owned the lowest levels of gingival index, plaque index, calculus index, PPD, and CAL, while GG genotype carriers had the highest values. It can be concluded that the periodontal status differed significantly among cases with different rs2569190 genotypes.

**Table 3:** Periodontal status in patients with *CD14* gene rs2569190 different genotypes.

Indicators	Genotype			*p* Value
	AA	AG	GG	
Gingival index	2.42 ± 0.50	2.48 ± 0.54	2.71 ± 0.49	.042*
Plaque index	2.09 ± 0.58	2.12 ± 0.65	2.46 ± 0.51	.014*
Calculus index	0.30 ± 0.47	0.48 ± 0.51	0.74 ± 0.70	.006**
PPD (mm)	5.06 ± 0.10	5.56 ± 0.78	5.60 ± 0.50	.007**
CAL (mm)	4.58 ± 0.71	4.62 ± 0.63	5.11 ± 0.53	<.001***

*Note*: The data are represented as mean ± SD.

Abbreviations: AA, Adenine‐Adenine; AG, Adenine‐Guanine; CAL clinical attachment level; GG, Guanine‐Guanine; PPD, peri‐implant pocket depth.

* Statistical difference with the *p*‐value less than .05.

** Statistical difference with the *p*‐value less than .01.

*** Statistical difference with the *p*‐value less than .001.

Similarly, values of the periodontal status‐related factors were also compared among different *CD14* gene rs2915863 groups. As shown in Table 4, no statistical significance in terms of gingival index, plaque index, calculus index, PPD, and CAL was detected among individuals carrying different genotypes (*p* > .05). The findings indicated that *CD14* gene rs2915863 polymorphism might be not related to periodontal status of PI patients.

**Table 4:** Periodontal status in patients with different genotypes of *CD14* gene rs2915863.

Indicators	Genotype			*p* Value
	TT	TC	CC	
Gingival index	2.35 ± 0.49	2.57 ± 0.54	2.57 ± 0.50	.223
Plaque index	2.00 ± 0.46	2.22 ± 0.68	2.27 ± 0.57	.224
Calculus index	0.50 ± 0.51	0.41 ± 0.50	0.61 ± 0.67	.228
PPD (mm)	5.55 ± 0.69	5.33 ± 0.77	5.49 ± 0.88	.469
CAL (mm)	4.75 ± 0.72	4.73 ± 0.61	4.76 ± 0.71	.975

*Note*: The data are represented as mean ± SD.

Abbreviations: CAL clinical attachment level; CC, cytosine–cytosine; PPD, peri‐implant pocket depth; TC, thymine–cytosine; TT, thymine–thymine.

## 4 DISCUSSION

PI is an infectious disease affecting the soft and hard osseous tissue surrounding the implants, which is the main cause of the failure of the implant denture.^15^ With the increasing demand for dental implants, the risk of dental implant failures appear to increase recently.^16^ Many factors can influence the occurrence of PI, including occlusal factors, general condition, smoking and oral health habit, and so on.^17^ When eliminated the possible risk factors, it is noted that most of PI occurs in a specific population, which is related to the host response to the bacterial challenge and susceptibility.^18^ Thus, host factor is essential in the occurrence of PI. Furthermore, patients had the history of one implant failure are much easier to failure of additional implants. And the susceptibility might be associated with genetic factors.^19^

CD14 is a major endotoxin receptor which is implicated in the phagocytosis of bacteria and LPS‐mediated bone resorption.^20^, ^21^ The CD14 expression can be activated by the stimulation of NF‐κB and consequential upregulation of transcription of genes coding various pro‐inflammatory cytokines.^22^ CD14 is a glyco‐phosphatidylinositol‐linked protein with the function of transferring LPS to toll‐like receptors. Additionally, CD14 can affect subsequent production of pro‐inflammatory cytokines, further resulting in LPS‐stimulated bone resorption.^23^ The human *CD14* gene is a single‐copy gene but encodes two distinctive protein forms. The membrane‐bound CD14 receptor protein is mainly expressed on the surface of monocytes, macrophages, neutrophils, and gingival fibroblasts, it can participate in the process of cellular response to bacterial LPS. The other form, known as soluble form, is detected in monocyte or liver but lacks the anchor.^24^ CD14 has been considered as a causative gene for periodontitis, and is implicated in periodontal tissue remodeling and degradation.^25^

In the current study, the *CD14* gene two common polymorphisms namely, rs2569190 and rs2915863, were analyzed in the Chinese Han population. The results determined that the control group had a lower frequency of rs2569190 GG genotype than the PI group did. And the distribution differences reached a significant level. However, the AG genotype frequency of rs2569190 was almost equal between the two groups. All data implied the significant association of *CD14* gene rs2569190 polymorphism with PI susceptibility in the Chinese Han population. And compared with AA genotype, the GG genotype carriers were much easier to suffer from PI. Rs2569190 polymorphism, also referred as CD14‐159C/T, is a functional SNP located in the 5′ UTR of the promoter region of *CD14* gene. It exerts considerable impact on the CD14 receptor, and then further influences the functionality of monocytes.^26^ As reported in a meta‐analysis, *CD14* ‐159C/T has been suggested to be involved in the development of periodontitis, which was in accordance with our present results.^27^ Furthermore, Rakic et al.^12^ have reported a significant association between *CD14* ‐159C/T polymorphisms and PI susceptibility, and rs2569190 may be a potential biomarker for PI, all evidence supported our present conclusion. In a study of sepsis, both C reactive protein and procalcitonin levels were higher among the AA homozygotes compared with the G‐allele carriers, indicating that a significantly stronger pro‐inflammatory response among AA patients compared with G‐allele carriers.^28^ But according to present findings, the GG genotype carriers were much easier to suffer from PI. Considering the low pro‐inflammatory response and high PI predisposition of GG genotype carriers, this may be attributed to immune suppression in individuals, which may be a risk factor for implant‐related infection.^29^ However, the assumption needs to be confirmed. Rs2915863, also known as CD14 G‐1619A, is another common SNP located on CD14 promoter region at position ‐1619.^30^ Rs2915863 polymorphism was also analyzed in this paper, but no significant association was detected with PI susceptibility in the Chinese Han population.

## 5 CONCLUSION

In conclusion, our results showed that the rs2569190 polymorphisms of *CD14* gene had a significant association with PI susceptibility in the Chinese Han population, and the GG genotype and G allele were risk factors for the occurrence of PI. Other studies were also needed to confirm our results due to the following reasons. First, a limited number of the study sample was recruited in this study, more eligible participants are feasible to reduce the risk of bias. Second, PI is a multifactorial disease, which is not affected by only one gene. Thus, other genes and polymorphisms should be considered. Thus other replication studies in various populations should also be performed. Additionally, an in‐depth mechanism analysis is lacking, which should be considered in future studies.

## AUTHOR CONTRIBUTIONS

**Jie Li**: Conceptualization; data curation; methodology; writing—original draft. **Xiao Qiao**: Data curation; formal analysis; investigation; resources; software. **Jin Shang**: Conceptualization; project administration; supervision; writing—review & editing.

## CONFLICT OF INTEREST STATEMENT

The authors declare no conflict of interest.

## Data Availability

The data sets used and/or analyzed during the current study are available from the corresponding author on reasonable request.
